# First person – Ionel Sandovici

**DOI:** 10.1242/dmm.052045

**Published:** 2024-08-29

## Abstract

First Person is a series of interviews with the first authors of a selection of papers published in Disease Models & Mechanisms, helping researchers promote themselves alongside their papers. Ionel Sandovici is first author on ‘ A genetically small fetus impairs placental adaptations near term’, published in DMM. He is a Senior Research Associate in the lab of Miguel Constância at The Institute of Metabolic Science, University of Cambridge, Cambridge, UK. Ionel investigates signals that enable the communication between the fetus, placenta and mother during intrauterine development, and the mechanisms through which these signals work.


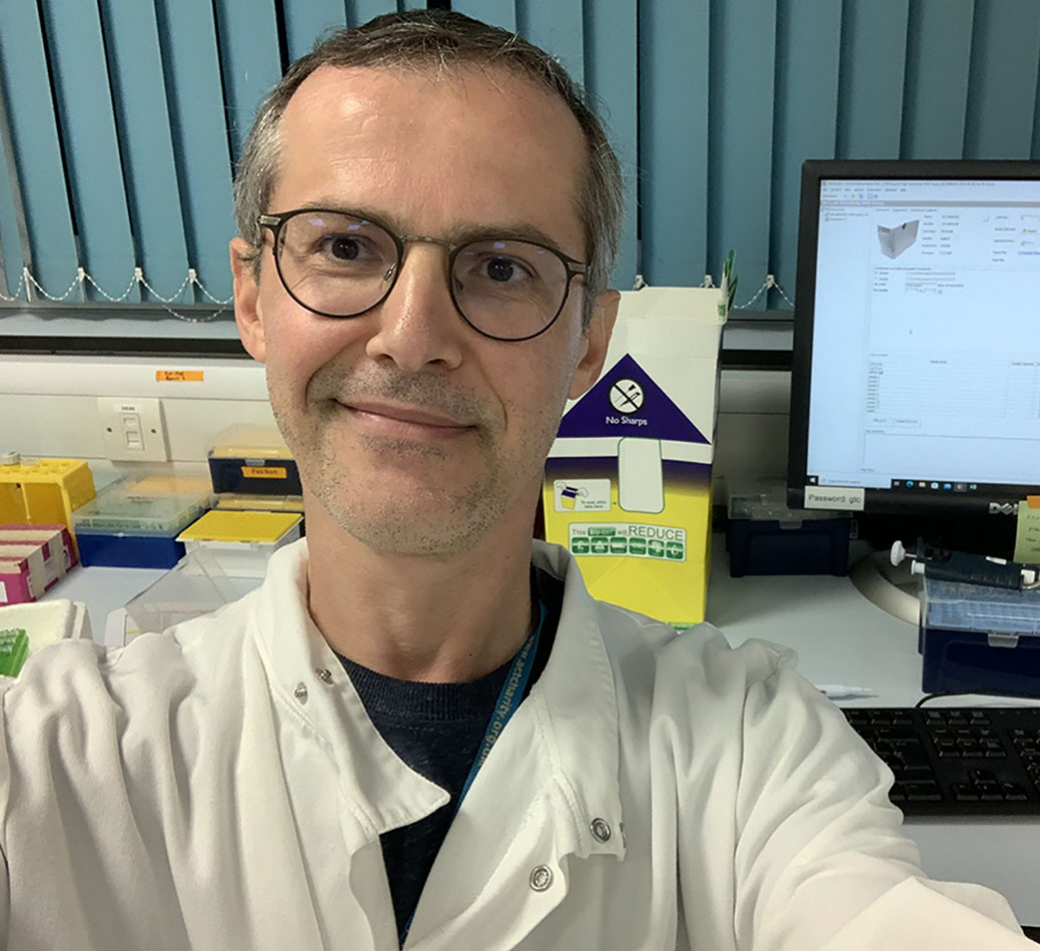


**Ionel Sandovici**

**Who or what inspired you to become a scientist?**

I started my career by studying for a medical degree in my home country, Romania. During the medical school I had amazing mentors, who opened by mind about the importance of fundamental research. I realized that what interests me the most is understanding ‘how our body works’, be that at physiological or at molecular level. Although I practiced medicine for a few years after finishing my medical training, I was constantly looking for opportunities in basic research. That led me to applying for a PhD, which allowed me to study abroad in the United States, at the Fels Institute in Philadelphia. Although I thought initially that I would like to combine medical practice with basic research, over time I concluded that being a scientist is a very fulfilling career that brings me a lot of joy and professional satisfaction.

Fetal growth restriction can lead to significant short-term and long-term complications, adversely impacting quality of life.

**What is the main question or challenge in disease biology you are addressing in this paper? How did you go about investigating your question or challenge?**

Although it affects 3–7% of all pregnancies, the pathophysiology of fetal growth restriction remains poorly understood. Fetal growth restriction can lead to significant short-term and long-term complications, adversely impacting quality of life. One of the major challenges is that fetal growth restriction is a complex condition with numerous fetal, placental, and maternal causes, often with significant overlap in their pathogeneses. In humans, it is often difficult to identify the original cause and to study the pathophysiological events in a temporal manner. Animal models of human diseases are very useful in this respect, as they allow specific manipulation and precise control over key factors that can impact the outcome. In our paper, we used a mouse model that had recently been developed in our lab (Sandovici et al., 2022), which enabled the deletion of a key growth-promoting gene, i.e. insulin-like growth factor 2 (Igf2), exclusively in the fetus. This model allowed us to carefully characterize how the placenta adapts during the latter stages of pregnancy when the fetal demand for growth is reduced.

**How would you explain the main findings of your paper to non-scientific family and friends?**

In this study, we explored how a fetus influences its placenta to ensure it gets the appropriate amount of nutrients needed for normal growth. We used a mouse model to focus on a specific gene called *Igf2* that – as previously demonstrated by us – helps the fetus to communicate its growth needs to the placenta. By removing this gene from the fetus and, thus, reducing its growth demands, we characterized the sequence of adaptations undertaken by the placenta during later stages of pregnancy. We found that the placenta undergoes structural changes leading to a less expanded nutrient-exchange area. Additionally, the transfer of essential nutrients, such as glucose and amino acids, from mother to fetus was reduced, especially at the end of pregnancy when the fetus normally grows very rapidly. This reduction was more pronounced in female fetuses. Furthermore, the blood flow to the placenta was reduced, similar to patterns seen in human pregnancies affected by fetal growth restriction.

[…] enhancing IGF2 in the placenta during the late stages of pregnancy potentially improves outcomes for growth-restricted fetuses, offering a new direction for therapeutic interventions.

**What are the potential implications of these results for disease biology and the possible impact on patients?**

Our study highlights the critical role played by IGF2 in fetal growth and placental function. We believe that enhancing IGF2 in the placenta during the late stages of pregnancy potentially improves outcomes for growth-restricted fetuses, offering a new direction for therapeutic interventions.

**Figure:**
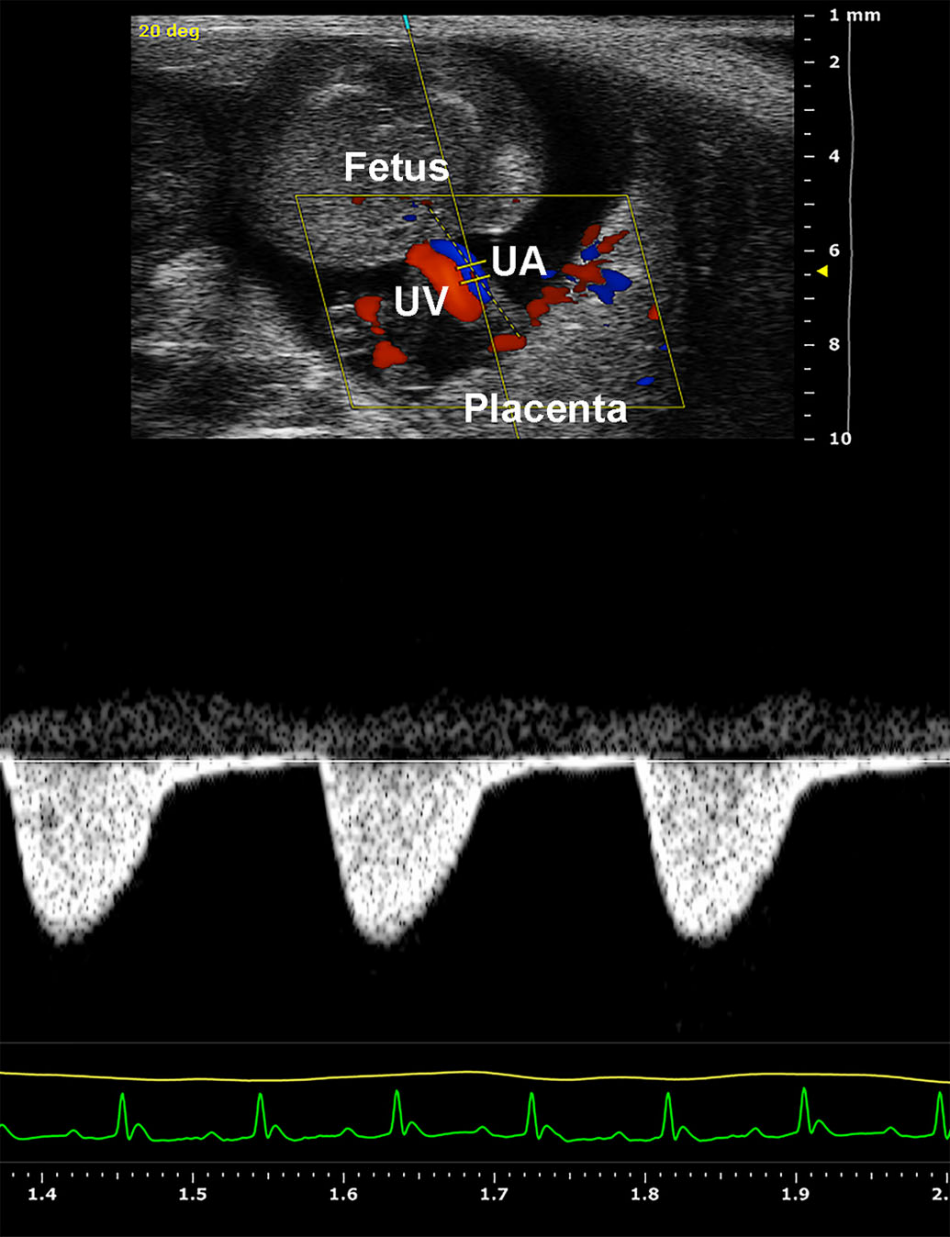
**Recording the blood waveforms of the umbilical artery in a mouse conceptus at E15.5 during pregnancy using Power Doppler ultrasonography.** UA, umbilical artery; UV, umbilical vein.

**Why did you choose DMM for your paper?**

I submitted this paper to DMM because it is a journal that aims to provide a platform appealing to a broad readership, including basic researchers, translational scientists and clinicians, fostering communication among them. Additionally, I appreciate the journal's ethos, as all articles are published without profit under an Open Access model, as well as its initiative to interview first authors of papers published in DMM. Finally, our university has a ‘Read & Publish Open Access’ agreement with The Company of Biologists, which includes DMM as part of the five-journal package.

**Given your current role, what challenges do you face and what changes could improve the professional lives of other scientists in this role?**

I believe that significant scientific discoveries require a long-term vision and a reduction in the aversion to risk. The standard 3-year postdocs are often too short for challenging projects, and the emphasis on ‘publish or perish’ has led the scientific community to adopt practices that are, ultimately, not beneficial for society. Although the scientific community and governments worldwide are working to address these challenges, there is still a long way to go. From my perspective, I would like to see funding bodies become less risk averse, particularly when supporting the younger generation of scientists through personal fellowships or smaller grants. Major scientific discoveries are often made by young individuals who, while they may have less experience than established experts, are more willing to try new approaches to important questions.

**What's next for you?**

For many years, I have been working on several projects simultaneously, each with diverse aims and approaches. I find this way of working enjoyable, as it adds dynamism to my professional life and provides opportunities to learn from and collaborate with a wide range of talented and intelligent individuals. One of the new directions emerging from the studies highlighted above is to uncover how the placenta, mother and fetus communicate at the molecular level to ensure a successful pregnancy. We are using transgenic mouse models to capture and identify the proteins that are exchanged between these compartments. Our longer-term goal is to elucidate their modes of action and to explore their potential as new therapeutic targets in order to alleviate complications of pregnancy.

**Tell us something interesting about yourself that wouldn't be on your CV**

If I hadn't studied medicine and become a scientist specializing in developmental biology and genetics, I would have liked to become a historian. I greatly enjoy learning about the legacies of past people and civilizations. I love reading history books and visiting historical sites around the world whenever I have the chance.
